# Sexual dimorphism in anxiety susceptibility: role of PNN maturation timing in the habenulo-interpeduncular reward circuits

**DOI:** 10.1016/j.ynstr.2025.100750

**Published:** 2025-08-17

**Authors:** Niels Fjerdingstad, Malalaniaina Rakotobe, Célia Leboulenger, Adrien Chopin, Thomas Lamonerie, Fabien D'Autréaux

**Affiliations:** Université Côte D’Azur, CNRS, Inserm, IBV, Institut de Biologie Valrose, 06108, Nice, France

## 1 Introduction

Critical periods are windows of development during which the brain is particularly plastic and sensitive to environmental influences (Sale et al., 2014). Disturbances and negative experiences during these periods, such as stress, in interaction with genetic and hormonal factors, can lead to lasting changes in brain structure and function, predisposing individuals to disorders such as depression and anxiety. It is the reason why these disorders are now considered to have a neurodevelopmental origin (Agorastos et al., 2019). In mammals, neurodevelopment extends into adulthood, with an intense phase in puberty. Here, profound hormonal changes lead to the maturation of reproductive systems and the expression of secondary sexual characteristics. These changes also trigger significant brain plasticity, influencing both the structure and function of the neural circuits that control behavior and cognition (Blakemore et al., 2010). In humans, it is suspected that stress during puberty can induce persistent changes in behavior and an increase in anxiety in adulthood, with a more marked incidence in women than in men (Holder and Blaustein, 2014). Puberty is therefore a critical period in brain development, with potential sexual dimorphism.

The search for the molecular and cellular basis of brain plasticity has highlighted the importance of specialized extracellular matrix structures called perineuronal nets (PNNs), particularly during critical periods (Wang and Fawcett, 2012). PNNs primarily surround fast-spiking parvalbumin-containing GABAergic interneurons and help stabilize their synaptic connections (Wingert and Sorg, 2021). This stabilization is essential for maintaining learned behaviors and memories, including those related to stress responses (Sanchez et al., 2024). However, PNNs behave differently in different brain regions, with some such as the primary visual cortex showing classical PNN-mediated closure (Pizzorusso et al., 2006), while others such as the hippocampus and prefrontal cortex, showing ongoing plasticity regulated by dynamic PNNs (Carulli and Verhaagen, 2021). This flexibility enables the brain to optimize both stability and adaptability, according to the specific functional needs of each region. If abnormal or excessive stress occurs during PNNs formation, it can leave a lasting imprint on neural circuits, essentially locking-in the changes induced by stress. How stress leaves this long-lasting imprint is not yet fully understood, but research suggests it involves a combination of synaptic changes and structural alterations in the PNNs themselves. Stress during the later stages of PNN formation can alter PNN structural integrity, either transiently or over the long-term. In some cases, these changes can lead to a reduction in PNN density (Pesarico et al., 2019). The degradation of perineuronal nets (PNNs), whether due to stress or enzymatic breakdown (e.g., with chondroitinase), generally leads to reduced spiking activity in parvalbumin (PV) neurons and increased overall excitability. Whether these changes are labile or fixed may also depend on the timing and severity of the stress, as well as the specific brain regions involved. Molecular players involved in PNN assembly, such as the homeoprotein Otx2, are also potential mediators of these effects, further adding to the complexity of how stress interacts with PNNs during development (Gibel-Russo et al., 2022).

We recently developed a peri-pubertal stress protocol that enabled us to incriminate a subpart of the habenulo-interpeduncular system (HIPS), a core component of reward circuits, in anxiety susceptibility (Rakotobe et al., 2024). This subpart, called the HIPOPS (Habenulo-InterPeduncular Otx2-Positive System), is composed of two interconnected structures: the more central part of the medial habenula (MHb), highly expressing the Otx2 homeodomain transcription factor, and interpeduncular nucleus (IPN) neurons also expressing Otx2. This HIPS subcircuit is entirely Otx2-dependent since deletion of the Otx2 gene interferes with both MHb and IPN development and activity (Ruiz-Reig et al., 2019). We have shown that chronic restraint stress at the onset of puberty in males makes these animals susceptible to develop anxiety whereas blocking HIPOPS activity during stress exposure prevents its early effects and promotes long-term resilience to anxiety (Rakotobe et al., 2024). In this study, we tested whether the same pubertal stress mechanisms responsible for anxiety susceptibility exist in females and whether these mechanisms involve PNNs located in the HIPOPS.

## 2 Materials and methods

### 2.1 Animals

All mice used in this study were bred and housed in the animal facility of the Institut de Biologie Valrose, Nice, France. All mouse lines were maintained on a 129/Sv genetic background. Both male and female mice were used in this study. Behavioral tests, restraint stress, and sacrifices were performed during the light phase of the 12-h light/dark cycle. All experimental protocols and procedures were conducted in compliance with European Union regulations and were approved by the local ethics committees and the French Ministry.

### 2.2 Stereotactic injections of ChABC

Animals were anesthetized via intraperitoneal (IP) injection of a mixture of Zoletil (4 mg/kg body weight), Rompun (6 mg/kg body weight), and Metacam (2 mg/kg body weight) (ZRM mixture) at a dosage of 10 μL/g body weight. The animal's scalp was shaved, and Xylocaine gel was applied to the skull before incision and in the ears to reduce pain caused by the ear bars of the stereotactic apparatus. After disinfecting the incision area, each animal was placed and secured in a stereotactic frame. Eyes were protected from drying with Ocry-gel. Coordinates were determined using the Paxinos and Franklin atlas (2004). The implantation was performed first on the left and then on the right in the IPN according to the following coordinates: A/P = −3.7; D/V = −5.0; L = ±0.25.

A total of 500 nl of ChABC (50 U/mL in PBS, Sigma-Aldrich C3667) or penicillinase (PCN; Sigma-Aldrich 61305) was injected at a rate of 100 nl/min. After the injection, the cannula was left in place for 5 min to allow diffusion before removal. At the end of the surgery, an anti-inflammatory drug (Metacam, 0.5 mg/mL) was administered via IP injection. Anti-inflammatory treatment continued for 2 days alongside post-operative monitoring of the animals.

### 2.3 Stress protocol

Mice were placed in a restraint device (Bio Services, 551B-BSRR) to minimize movement. This restraint was applied chronically (2h/day, 7 consecutive days). For analyses of neuronal activity at different developmental stages, animals were immediately sacrificed at the end of the final chronic stress session. Control animals were not exposed to stress and were sacrificed at the appropriate age.

### 2.4 Behavioral tests

Animals were acclimated for 1 h in the testing room before the start of behavioral experiments. Arenas were pre-cleaned with 70 % ethanol before each mouse was introduced. Video recordings were acquired and analyzed using EthoVision XT software.

a. Open Field Test

The open field test was used to assess anxiety levels and locomotor behavior in mice. The test was conducted in a black PVC arena with dimensions (40 L x 40 W × 30 H cm), divided into a peripheral zone (50 % of the total area) and a central zone (50 % of the total area). Mice were individually placed in a corner of the arena, and their behavior was recorded for 10 min. Only the first 5 min were analyzed to avoid habituation to the new environment. Measured parameters included the time spent in the center, number of entries into the center, maximal speed, and total distance traveled.

b. Elevated Plus Maze Test

The elevated plus maze test was used to assess anxiety levels. The apparatus consisted of two open arms (50 × 5 cm), which induce anxiety in mice, and two closed arms (50 L x 5 W × 16 H cm) extending from a central platform (5 × 5 cm), elevated 50 cm above the ground. Mice were placed at the center of the platform facing a closed arm, and behavior was recorded for 10 min. Measured parameters included the time spent, number of entries, the maximal speed, and total distance traveled in the open arms.

### 2.5 Histology and immunofluorescence

Animals were sacrificed immediately after the final stress session or without stress between 12pm and 4pm via a lethal IP injection of the ZRM anesthetic mixture (20 μL/g body weight), followed by intracardiac perfusion with PBS, then PBS-4 % paraformaldehyde (PFA). Brains were dissected and stored in 4 % PFA for 24 h at 4 °C for post-fixation, then rinsed three times and stored in PBS containing 0.05 % sodium azide until histological sectioning.

Brains were embedded in 4 % agar dissolved in PBS with 0.05 % sodium azide, kept in a liquid state at 50 °C, and then solidified at 4 °C for 5 min. The agar blocks were mounted and cut into 40 μm coronal sections using a Leica VT1000 S vibratome. Sections were stored in PBS with 0.05 % sodium azide until used for immunofluorescence staining.

Sections were permeabilized in PBS with 0.2 % Triton X-100 (Tx) and washed three times for 10 min each. They were then incubated in a blocking solution (PBS, 10 % Normal Donkey Serum, 0.2 % Tx) for 1 h. Tissues were incubated overnight at 4 °C with primary antibodies diluted in blocking solution. The next day, sections were washed and incubated for 2 h with secondary antibodies diluted in blocking solution. They were then washed again, incubated in DAPI (1 μg/mL), briefly rinsed in water to remove salt crystals, and mounted in Mowiol 4–88 on Superfrost + slides (Fisher Scientific).

Primary antibodies and lectin used: Rabbit anti-Egr1 (Cell Signaling; 1/1600), Goat anti-Otx2 (R&D Systems; 1/500), Biotinylated WFA (Vector Laboratories; 1/1000), Rabbit anti-Aggrecan (Sigma-Aldrich; 1/500). Secondary antibodies used (Jackson ImmunoResearch; 1/800): Anti-rabbit Alexa Fluor™-647, Anti-goat-Cy3, FITC-conjugated Streptavidin.

### 2.6 Microscopy and immunoreactivity measurements

Images were acquired using a wide-field AxioObserver – Zeiss microscope equipped with an ANDOR Neo sCMOS camera. The images were processed using FIJI software, and figures were assembled with Microsoft PowerPoint.

For Egr1 measurements, the mouse brain atlas was used as a reference to delineate the different subregions of the IPN. Three 40 μm-thick sections per animal were selected every two sections (bregma −3.4 to −3.88 mm) to cover the entire Otx2+ surface of the IPN. The same analysis protocol was then applied to all Egr1 measurements using a macro designed to standardize the measurements.

Briefly, the macro measures the background signal for both Egr1 and Otx2, then applies a threshold to the fluorescent intensity of the Egr1 signal, where the minimum parameter is determined based on the Egr1 background measurement. The binarized image is then processed with the “Analyze Particles” function in FIJI to create a mask containing Egr1+ cells detected above the configured threshold. This mask is then integrated as a Region of Interest (ROI) and used as an overlay to measure Egr1 and Otx2 signal intensities in the original image. The fluorescence intensity measurements for Egr1 and Otx2 from each Egr1+ cell are recorded in an Excel file, where Otx2+ and Otx2-cells are filtered based on the intensity of the Otx2 signal relative to background noise. The Egr1 immunoreactivity parameter presented in the data represents both area and fluorescence intensity of Egr1+ cells.

For PNN (Perineuronal Nets) analysis, two 40 μm-thick sections per animal were selected every two sections (bregma −3.4 to −3.88 mm) to cover the entire Otx2+ and WFA + surface of the IPN. The same analysis protocol was then applied to all WFA measurements using a macro designed to standardize the process.

Background noise was smoothed using the “Subtract Background” function with a rolling value of 50. Contrast was then enhanced using the “Unsharp Mask” function with a radius of 3 and a mask of 0.6. A background measurement was performed to apply a threshold, where the minimum parameter was set based on the background measurement of the marker of interest. The binarized image was then processed using FIJI's “Analyze Particles” function to create a mask containing positive signals for the marker of interest above the configured threshold. This mask was then integrated as a ROI and used as an overlay to measure the fluorescence intensities of WFA in the original image.

The measured parameters included the immunoreactivity area and intensity, where intensity represents the area factorized by the signal intensity normalized to background noise.

### 2.7 Statistical analysis

Statistical analyses were performed using R software and GraphPad Prism. Graphs were generated using Graph Robot tool (Lei A. Wang, 2019). Comparisons were made between different conditions of stress, age, or stereotactic injections within the same sex. Results are presented as mean ± SEM.

Data normality was assessed using the Shapiro-Wilk test. For comparisons between two groups, if the assumption of normality was violated, the non-parametric Mann–Whitney *U* test was used; otherwise, an independent samples *t*-test was applied. The F-test was used to assess the homogeneity of variances, and Welch's correction was applied when variances were unequal. For comparisons involving three groups, the Kruskal–Wallis test was used when data did not meet normality assumptions, followed by pairwise Mann–Whitney U tests as post hoc analysis. In this case, the Bonferroni correction was applied to adjust for multiple comparisons. If the normality and homogeneity assumptions were met, a one-way ANOVA was performed, followed by Tukey's HSD and LSD post hoc tests to determine group differences. A two-way ANOVA was used to determine if the factor sex was statistically different and if there is an interaction between stress and sex. A one-sample *t*-test was used to determine if the ratio CS/NS significantly differs from 1 showing if there is a significant effect of stress.

The global score was calculated as follows: For each parameter, the measured values of each animal in both the control and stressed groups were expressed as a percentage of the maximum value obtained across all animals in both groups. Then, the average percentage per animal across all parameters was computed. The final score for each animal corresponds to 100 % minus this average percentage.

Statistical details are presented in Table 1.

**Table 1:** Detailed statistics for all the figures.

Fig.1										
Test	OF Dist Center		OF Time Center		OF N°entries Center		OF Dist Center		OF Time Center	
Gender and Condition	M NS	M CS	M NS	M CS	M NS	M CS	F NS	F CS	F NS	F CS
N	17	12	17	12	17	12	14	9	14	9
Shapiro-Wilk (p value)	0.04052	0.119	0.07317	0.408	0.06498	0.8129	0.7506	0.7134	0.5938	0.3511
F test NS vs CS (p value)			0.0948		0.0223		0.2218		0.2277	
T test NS vs CS (p value)			0.0022		0.01412		0.1917		0.3672	
Mann-Whitney U NS vs CS (p value)	0.03572									

Test	EPM Dist Center		EPM N°entries Center		EPM Time Center		EPM N°entries Center		EPM Dist Center		EPM Time Center	
Gender and Condition	M NS	M CS	M NS	M CS	M NS	M CS	F NS	F CS	F NS	F CS	F NS	F CS
N	19	14	17	12	17	12	14	9	14	9	14	9
Shapiro-Wilk (p value)	0.6785	0.159	0.7701	0.2037	0.9572	0.5138	0.9346	0.5585	0.1557	0.05029	0.1407	0.3992
F test NS vs CS (p value)	0.3736		0.9353		0.1225		0.7864		0.8156		0.2408	
T test NS vs CS (p value)	0.0284		0.0274		0.1036		0.0613		0.6631		0.2698	

Test	Vmax			
Gender and Condition	M NS	M CS	F NS	F CS
N	19	14	14	9
Shapiro-Wilk (p value)	0.4626	0.9381	0.167	0.7494
F test NS vs CS (p value)	0.1354		0.1005	
T test NS vs CS (p value)	0.1777		0.3650	

Test	Global anxiety score			
Gender and Condition	M NS	M CS	F NS	F CS
N	19	14	14	9
Shapiro-Wilk (p value)	0.5757	0.439	0.1946	0.7164
F test NS vs CS (p value)	0.1905		0.8749	
T test NS vs CS (p value)	0.0006		0.2520	

Fig. 2				
Egr1 immunoreactivity % of background intensity				
Gender, Condition, Age	M NS P36	M CS P36	F NS P36	F CS P36
N	5	5	4	4
Shapiro-Wilk (p value)	0.9311	0.9875	0.8206	0.1069
Two-way Anova (p value)	Factor1 NS vs CS p = 0.6563; Factor2 M vs F p = 0.0139; Factor1 x 2 p = 0.0356			

Egr1 immunoreactivity % of background intensity												
Gender, Condition, Age	M NS P29	M CS P29	M NS P36	M CS P36	M NS P66	M CS P66	F NS P29	F CS P29	F NS P36	F CS P36	F NS P66	F CS P66
N	6	6	5	5	3	5	6	6	4	4	4	5
Shapiro-Wilk (p value)	0.2668	1	0.9311	0.9875	0.8206	0.1069	0.6812	0.156	0.8206	0.1069	0.2237	0.1562
F test NS vs CS (p value)	0.0008		0.7280		0.2706		0.0008		0.3142		0.0323	
T test NS vs CS (p value)			0.0043		0.9967				0.4047			
Mann-Whitney U NS vs CS (p value)	0.0242						0.0183				0.5763	

Egr1 immunoreactivity CS/NS						
Gender, Condition, Age	M CS/NS P29	M CS/NS P36	M CS/NS P66	F CS/NS P29	F CS/NS P36	F CS/NS P66
N	6	5	5	6	4	5
Shapiro-Wilk (p value)	1	0.9295	0.9875	0.156	0.1069	0.1562
One-way Anova (p value)	0.0151			0.0032		
Tukey HSD (p value)	0.9962 (P29 vs. P36); 0.0249 (P29 vs. P66); 0.0273 (P36 vs. P66)			0.0094 (P29 vs. P36); 0.0065 (P29 vs. P66); 0.9997 (P36 vs. P66)		
LSD (p value)	0.9351 (P29 vs. P36); 0.0099 (P29 vs. P66); 0.0108 (P36 vs. P66)			0.0036 (P29 vs. P36); 0.0025 (P29 vs. P66); 0.9805 (P36 vs. P66)		

Fig. 3						
WFA immunoreactivity intensity						
Gender, Condition, Age	M P29	M P36	M P66	F P29	F P36	F P66
N	6	5	3	6	4	3
Shapiro-Wilk (p value)	0.9502	0.9859	0.5121	0.04525	0.9314	1
One-way Anova (p value)	0.0025					
Tukey HSD (p value)	0.0020 (P29 vs. P36); 0.0643 (P29 vs. P66); 0.4040 (P36 vs. P66)					
LSD (p value)	0.0008 (P29 vs. P36); 0.0269 (P29 vs. P66); 0.2075 (P36 vs. P66)					
Kruskal–Wallis (p value)				0.0136		
Mann-Whitney U (p value)				0.0339 (P29 vs. P36); 0.0077 (P29 vs. P66); 0.5013 (P36 vs. P66)		

WFA immunoreactivity area						
Gender, Condition, Age	M P29	M P36	M P66	F P29	F P36	F P66
N	6	5	3	6	4	3
Shapiro-Wilk (p value)	0.9999	0.8231	0.6542	0.1522	1	0.9988
One-way Anova (p value)	0.0008			0.0056		
Tukey HSD (p value)	0.0008 (P29 vs. P36); 0.0204 (P29 vs. P66); 0.4689 (P36 vs. P66)			0.0674 (P29 vs. P36); 0.0054 (P29 vs. P66); 0.2707 (P36 vs. P66)		
LSD (p value)	0.0003 (P29 vs. P36); 0.0081 (P29 vs. P66); 0.2496 (P36 vs. P66)			0.0285 (P29 vs. P36); 0.0021 (P29 vs. P66); 0.1299 (P36 vs. P66)		

Fig. 4				
WFA immunoreactivity intensity				
Gender, Condition, Age	M NS P36	M CS P36	F NS P36	F CS P36
N	5	4	4	4
Shapiro-Wilk (p value)	0.9859	0.08887	0.9314	0.3848
**Two-way Anova (p value)**	Factor1 NS vs CS p = 0.0021; Factor2 M vs F p = 0.0442; Factor1 x 2 p = 0.0173			

WFA immunoreactivity area				
Gender, Condition, Age	M NS P36	M CS P36	F NS P36	F CS P36
N	5	4	4	4
Shapiro-Wilk (p value)	0.8231	0.4476	1	0.08586
**Two-way Anova (p value)**	Factor1 NS vs CS p = 0.0038; Factor2 M vs F p = 0.0665; Factor1 x 2 p = 0.0277			

WFA immunoreactivity intensity CS/NS						
Gender, Condition, Age	M CS/NS P29	M CS/NS P36	M CS/NS P66	F CS/NS P29	F CS/NS P36	F CS/NS P66
N	6	4	4	6	4	5
Shapiro-Wilk (p value)	0.1389	0.03887	0.1802	0.3109	0.3848	0.1288
One sample *t*-test (p value)	0.005	0.0025	0.2007	0.04621	0.195	0.4125
One-way Anova (p value)				0.1116		
Tukey HSD (p value)				0.7640 (P29 vs. P36); 0.0972 (P29 vs. P66); 0.3815 (P36 vs. P66)		
LSD (p value)				0.4930 (P29 vs. P36); 0.0415 (P29 vs. P66); 0.1928 (P36 vs. P66)		
Kruskal–Wallis (p value)	0.007					
Mann-Whitney U (p value)	0.0105 (P29 vs. P36); 0.0550 (P29 vs. P66); 0.0209 (P36 vs. P66)					

WFA immunoreactivity area CS/NS						
Gender, Condition, Age	M CS/NS P29	M CS/NS P36	M CS/NS P66	F CS/NS P29	F CS/NS P36	F CS/NS P66
N	6	4	4	6	4	5
Shapiro-Wilk (p value)	0.1351	0.4476	0.9433	0.8992	0.08586	0.2436
One sample *t*-test (p value)	0.1289	0.005183	0.104	0.04584	0.2776	0.4476
One-way Anova (p value)	0.0237			0.0817		
Tukey HSD (p value)	0.0416 (P29 vs. P36); 0.9081 (P29 vs. P66); 0.0328 (P36 vs. P66)			0.5946 (P29 vs. P36); 0.0680 (P29 vs. P66); 0.4252 (P36 vs. P66)		
LSD (p value)	0.0170 (P29 vs. P36); 0.6826 (P29 vs. P66); 0.0133 (P36 vs. P66)			0.3400 (P29 vs. P36); 0.0283 (P29 vs. P66); 0.2202 (P36 vs. P66)		

Fig. 5				
Egr1 immunoreactivity % of background intensity				
IPN region	cIPC + med-cIPL		Lat-cIPL	
Gender, Condition	M CS PCN	M CS ChABC	M CS PCN	M CS ChABC
N	9	9	8	8
Shapiro-Wilk (p value)	0.04681	0.7579	0.2008	0.9968
F test NS vs CS (p value)			0.8703	
T test NS vs CS (p value)			0.0790	
Mann-Whitney U NS vs CS (p value)	0.0086			

Fig. 6												
Test	OF Dist Center				OF Time Center				OF N°entries Center			
Gender and Condition	M CS PCN	M CS ChABC	F CS PCN	F CS ChABC	M CS PCN	M CS ChABC	F CS PCN	F CS ChABC	M CS PCN	M CS ChABC	F CS PCN	F CS ChABC
N	9	9	8	8	9	9	8	8	9	9	8	8
Shapiro-Wilk (p value)	0.1044	0.3705	0.2008	0.9968	0.0022	0.2135	0.8322	0.9995	0.0568	0.2395	0.6914	0.9411
F test NS vs CS (p value)	0.4518		0.0900				0.0740		0.6355		0.4404	
T test NS vs CS (p value)	0.0228		0.1155				0.0469		0.0401		0.1445	
Mann-Whitney U NS vs CS (p value)					0.4797							

Test	EPM Dist Center				EPM Time Center				EPM N°entries Center			
Gender and Condition	M CS PCN	M CS ChABC	F CS PCN	F CS ChABC	M CS PCN	M CS ChABC	F CS PCN	F CS ChABC	M CS PCN	M CS ChABC	F CS PCN	F CS ChABC
N	9	9	8	8	9	9	8	8	9	9	8	8
Shapiro-Wilk (p value)	0.8873	0.9579	0.4765	0.1498	0.9519	0.2658	0.9964	0.6818	0.5790	0.9602	0.5089	0.879
F test NS vs CS (p value)	0.0825		0.4598		0.7891		0.5619		0.1046		0.1994	
T test NS vs CS (p value)	0.0941		0.4664		0.2948		0.1618		0.1282		0.4301	

Test	Vmax			
Gender and Condition	M CS PCN	M CS ChABC	F CS PCN	F CS ChABC
N	9	9	8	8
Shapiro-Wilk (p value)	0.0001	0.0508	0.0061	0.0026
Mann-Whitney U NS vs CS (p value)	0.6048		0.8048	

Test	Global anxiety score			
Gender and Condition	M CS PCN	M CS ChABC	F CS PCN	F CS ChABC
N	9	9	8	8
Shapiro-Wilk (p value)	0.9506	0.1614	0.1385	0.6732
F test NS vs CS (p value)	0.4294		0.8000	
T test NS vs CS (p value)	0.0028		0.0315	

## 3 Results

1. Females do not exhibit the same response to peripubertal chronic stress

Previous research established that males subjected to moderate chronic stress (MCS) during the peripubertal period (P30-36), composed of 2 h of restraint stress every day for a period of 7 days followed by an identical chronic stress at the adult stage (P60-66), develop significant anxiety-related behaviors compared to non-stressed controls or controls that have only endured the adult stress (P60-66) (Rakotobe et al., 2024).

**Fig. 1:**
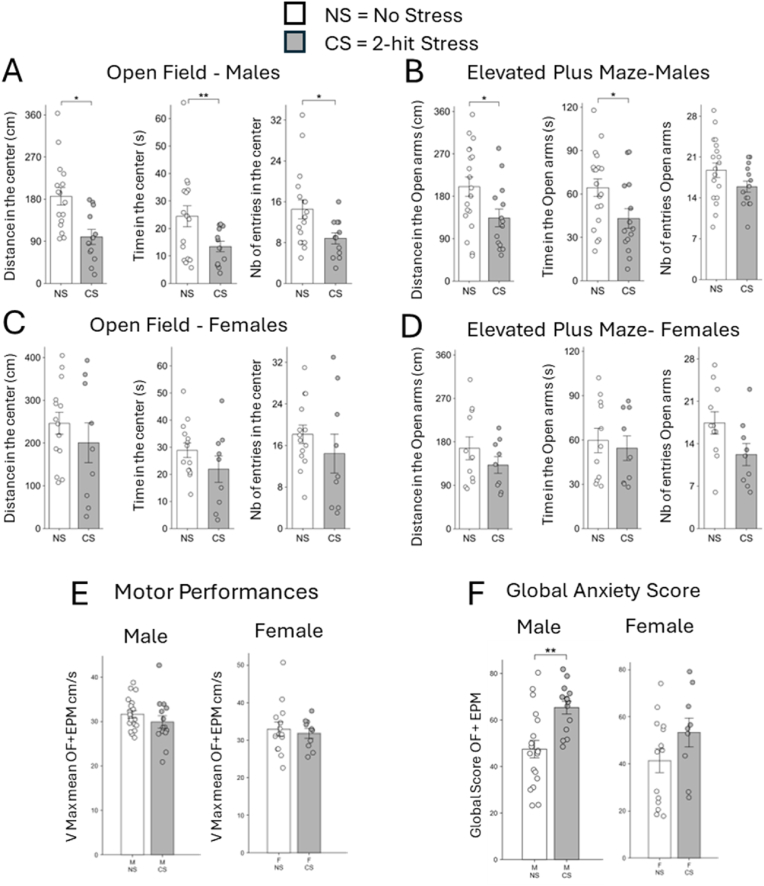
**Anxiety levels of male and female mice in OF and EPM behavioral tests under the 2-hit stress Protocol**. (A–D) Comparison of anxiety levels in male (A-B; NS: n = 17; 2xCS: n = 12) and female (C-D; NS: n = 14; 2xCS: n = 9) mice in the Open Field (A,C) and Elevated Plus Maze (EPM) tests (B,D). In the OF and EPM tests, the following parameters were measured: Distance traveled, time spent and number of entries in the center (A,C) or in the open arms (B,D). Motor performance was assessed by averaging the maximum velocity observed in the OF and EPM tests (E). An anxiety ranking score was calculated based on the different parameters (F). Data are presented as mean ± SEM. ∗P < 0.05; Mann-Whitney *U* test for comparison of distance in the center in (A) and ∗P < 0.05, ∗∗P < 0.01; two-tailed *t*-test for all other parameters. See Table 1 for statistical details.

To test whether the same peripubertal stress triggers anxiety susceptibility in females, we compared males and females subjected to the identical 2-hit chronic stress protocol described above and assessed behavior using the open field (OF) and elevated plus maze (EPM), two tests commonly used to evaluate anxiety-like behavior. As expected, males subjected to the 2-hit stress (Fig. 1A–B; CS) exhibited higher levels of anxiety compared to the control group (Fig. 1A–B; NS). This was evident for all the parameters measured in the tests, except for the number of entries in the EPM (Fig. 1B, right graph), which nevertheless showed the same trend. For females, the results showed a trend towards increased anxiety in the CS group, but the observed differences were not statistically significant (Fig. 1C–D). Females showed a broader distribution of anxiety scores, suggesting a higher variability in response to the 2-hit stress protocol. This phenomenon was particularly conspicuous in the OF test (Fig. 1C). This higher variability in females compared to males might be attributed to a subset of females displaying resistance to the stress protocol, as shown by the more dispersed data points in the female CS group across the various behavioral measures. The overall global anxiety scores (Fig. 1E), consolidated multiple behavioral measures, further reinforcing the statistical significance of these findings only in males (p < 0.001). We analyzed motor performance in male (Fig. 1F; left graph) and female mice (Fig. 1F; right graph) across the tests by recording maximal speed of each animal across the 5 min test and then calculating the mean between OF and EPM tests. No differences were detected, demonstrating the lack of motor dysfunctions that could have accounted for the decreased frequency, time and distance in the two tests.

Together, these results suggest a gender-specific impact of chronic stress on anxiety, with males being more uniformly affected by the 2-hit stress protocol than females.

2. IPN response to stress is different in females and dependent on the period considered

**Fig. 2:**
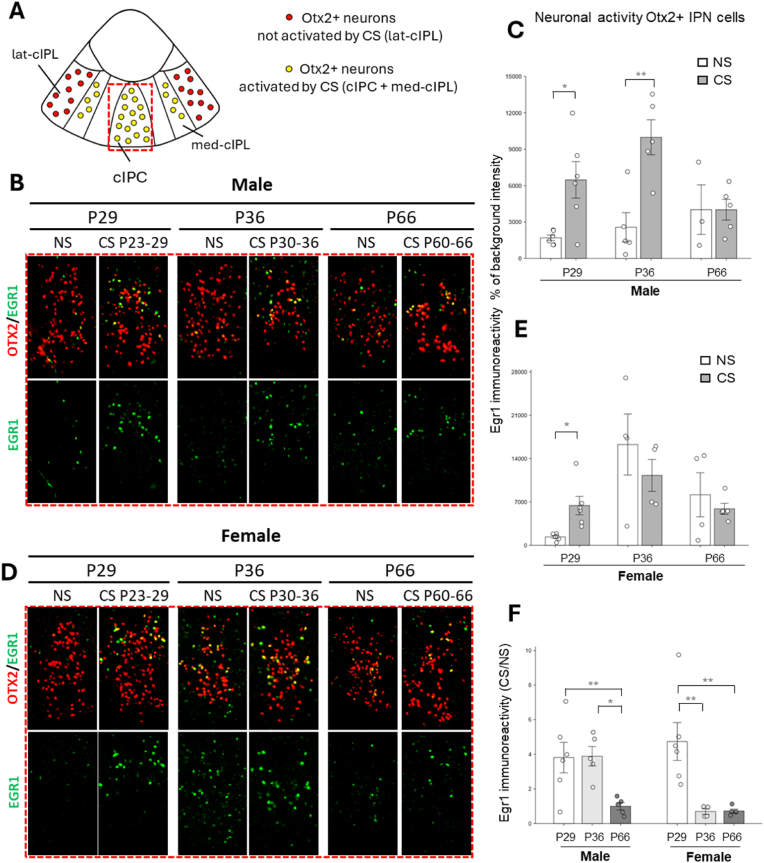
**Activity of Otx2+ neurons in the cIPC + med-cIPL region in response to CS at different postnatal periods in male and female mice.** (A) Schematic representation of a coronal section of the IPN, highlighting the cIPC + med-cIPL subregions containing CS-sensitive Otx2+ neurons (yellow) and the lat-cIPL subregions containing CS-insensitive Otx2+ neurons (red). The dashed red rectangle indicates the region shown in subfigures B and D. (B, D) Immunofluorescence labeling of coronal IPN sections for Otx2 (red) and Egr1 (green) in control (NS) and stressed (CS) male (B) and female (D) mice at P29, P36, and P66. Scale bar = 100 μm. (C, E) Quantification of Otx2+ neuronal activity based on Egr1 immunoreactivity, expressed as a percentage of background intensity, in the cIPC + med-cIPL region of control (NS) and stressed (CS) male (C) and female (E) mice at P29, P36, and P66. (F) Ratio CS/NS of the measures shown in C and E. Males: P29 NS (n = 6), P23-29 CS (n = 6), P36 NS (n = 5), P30-36 CS (n = 5), P66 NS (n = 3), P60-66 CS (n = 5). Females: P29 NS (n = 6), P23-29 CS (n = 6), P36 NS (n = 4), P30-36 CS (n = 4), P66 NS (n = 4), P60-66 CS (n = 5). Error bars represent SEM. ∗P < 0.05; ∗∗P < 0.01; two-tailed *t*-test between NS and CS conditions (C,E). ∗P < 0.05; ∗∗P < 0.01; One-way ANOVA and LSD (Least Significant Difference) test by Fisher as a post hoc test (F).

We then wanted to check whether the female IPN responded to the first chronic stress of the 2-hit stress protocol in the same way as the male IPN. The period of application of this first stress corresponds to the period of maximal response of Otx2+ IPN neurons to this stress in males, measured by the increase in expression of the neuronal activation marker Egr1. We thus subjected males and females to stress between P30 and P36 and examined Egr1 expression in the IPN regions previously shown to be activated by peripubertal chronic stress (Rakotobe et al., 2024) (Fig. 2A). As expected, stress applied between P30 to P36 significantly increased Egr1 expression in Otx2+ IPN neurons in males (Fig. 2B–C; see CS P30-36 vs NS condition). In sharp contrast in females, activity of the IPN was already high in controls at P36 and no further increase could be detected when chronic stress was applied at P30-36 (Fig. 2D–E; see CS P30-36 vs NS condition). As a result, the ratio between Egr1 immunoreactivity in the CS and NS conditions was significantly higher in males (Fig. 2F; see P36 for males and females).

These data suggested that, in females, the IPN has a higher baseline activity and is less sensitive to stress compared with males at least at P36. Given that the stress response in the IPN appears to peak in males around the onset of puberty, it is also plausible that a significant stress response in females occurs at a different developmental stage, which may correspond to the early onset of puberty, reported at P26, in females (Bell, 2018). To explore this hypothesis, we applied chronic stress earlier, at P23-29. Additionally, we applied stress after adolescence (P60-66) to test how females cope with stress once puberty is over. Stress applied at P23-29 strongly increased Egr1 expression in both males and females, whereas stress applied after adolescence had no impact on Egr1 expression in both sexes (Fig. 2B–F).

These results demonstrate that the responsivity of Otx2+ IPN neurons to moderate chronic stress in females decreases earlier than in males, suggesting a sex-specific difference in the timing of HIPOPS circuit plasticity. Furthermore, the higher baseline activity and the lack of IPN sensitivity to stress of females between P30–36 may explain why they did not develop anxiety related behavior when subjected to the 2-hit stress protocol (Fig. 1).

3. PNN maturation stabilizes by P36 in both male and female mice

The maturation of perineuronal nets (PNNs) is a hallmark of critical periods, when circuit plasticity is maximal. Our previous research has shown that PNNs, marked by the classical marker Wisteria floribunda agglutinin (WFA), surround Otx2+ neurons in the IPN (Rakotobe et al., 2024). This population of Otx2+ neurons is both GABAergic (Ruiz-Reig et al., 2019) and parvalbumin positive (Rakotobe et al., 2024). On this basis, we hypothesized that PNNs could influence anxiety susceptibility by imprinting stress-induced hyperactivation in Otx2+ IPN neurons during a critical period. For this hypothesis to hold true, the period of stress sensitivity must correspond to the period of PNN maturation in the IPN of male and female subjects.

**Fig. 3:**
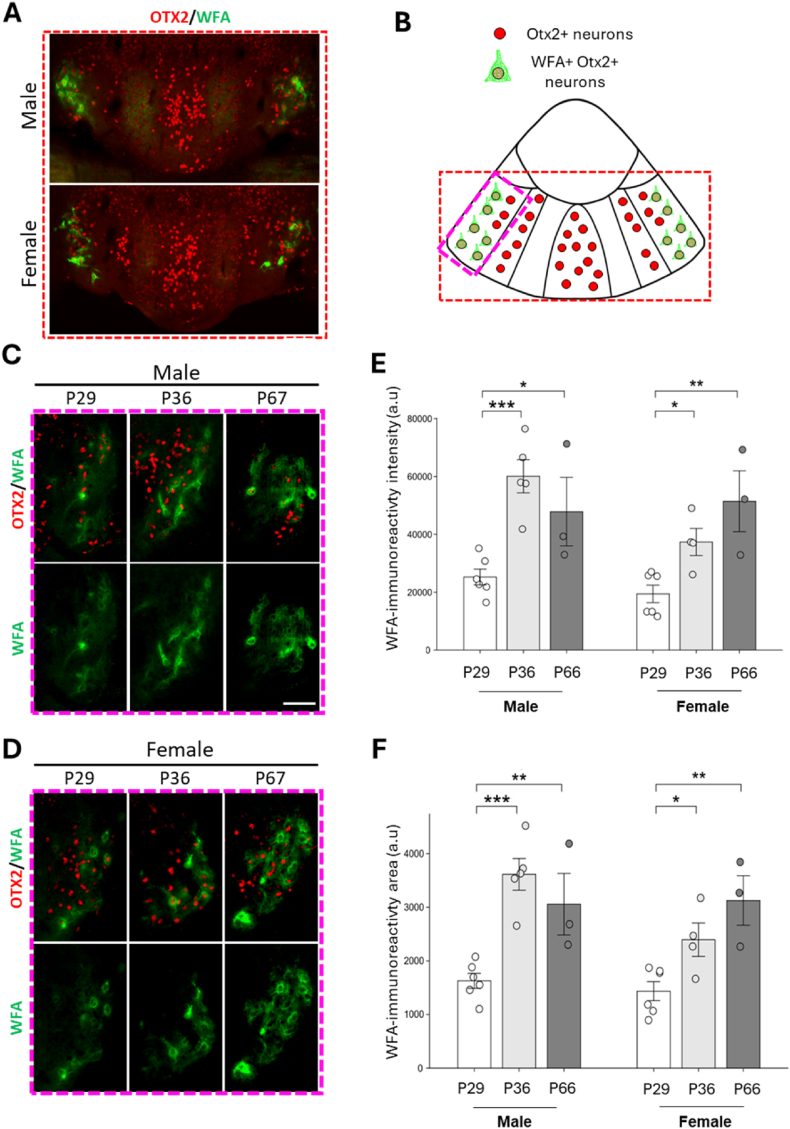
**Analysis of PNNs around Otx2+ neurons in the IPN at different postnatal stages in male and female mice.** (A) Immunostaining of coronal sections of the caudal IPN (cIPN) with anti-Otx2 antibodies (in red) and WFA (in green) in adult female and male mice. Scale bar: 100 μm. (B) Schematic illustration of the caudal IPN showing Otx2+ neuronal populations surrounded by WFA + PNNs (in green), in the lateralmost parts of the lateral IPN (lat-cIPL). The red and pink dashed rectangles highlight, respectively, the region shown in panel A, and the subregion shown in panels C and D. (E, F) Quantification of WFA labeling intensity (E) and surface area (F) in female and male mice at ages P29 (female: n = 6; male: n = 6), P36 (male: n = 5; female: n = 4), and P66 (female: n = 3; male: n = 3). ∗P < 0.05; ∗∗P < 0.01; ∗∗∗P < 0.001; One-way ANOVA and LSD (Least Significant Difference) test by Fisher as a post hoc test. ∗P < 0.05; ∗∗P < 0.01; Kruskal–Wallis and Mann-Whitney U as a post hoc test (E, female).

In the HIPOPS WFA + cells were only detected in the IPN (Fig. 3) but not in the MHb (Supplemental Fig. 1). To monitor PNN maturation in the IPN, we analyzed WFA staining, at three distinct postnatal stages: P29, P36, and P66. Separate WFA intensity and area analyses were performed for male and female subjects to assess developmental changes and potential sex differences in PNN maturation.

In males and females, WFA staining was found surrounding Otx2+ IPN neurons exclusively in the lateral regions of the IPN (Fig. 3A–B). There was a significant increase in maturation or increased density of PNNs around neurons during this developmental period. However, no significant differences were observed between P36 and P66, indicating that WFA intensity stabilizes by P36 in both sexes.

Similarly, there was a significant increase in WFA area from P29 to P36 (Fig. 3C, D, F), suggesting an expansion of PNN coverage around neurons. This increase in area may reflect active growth or spreading of PNNs during early postnatal development. By P66, no significant changes in WFA area were observed compared to P36, indicating that the physical extent of PNNs reaches a plateau by this stage.

These results indicate that WFA-labeled PNNs undergo significant maturation between P29 and P36, with an increase in both intensity and area. This period of development seems critical for the consolidation of PNNs around neurons. At P66, WFA intensity and area stabilize at identical levels in males and females, suggesting that PNN maturation reaches a steady state. The patterns observed in males and females suggest a conserved trajectory of PNN development between the sexes, with some variation in the extent of change during the early postnatal stages. A trend is observed in males, indicating that PNN maturation reaches a plateau earlier than in females.

4. Sex-Specific Effects of Chronic Stress on PNN development

**Fig. 4:**
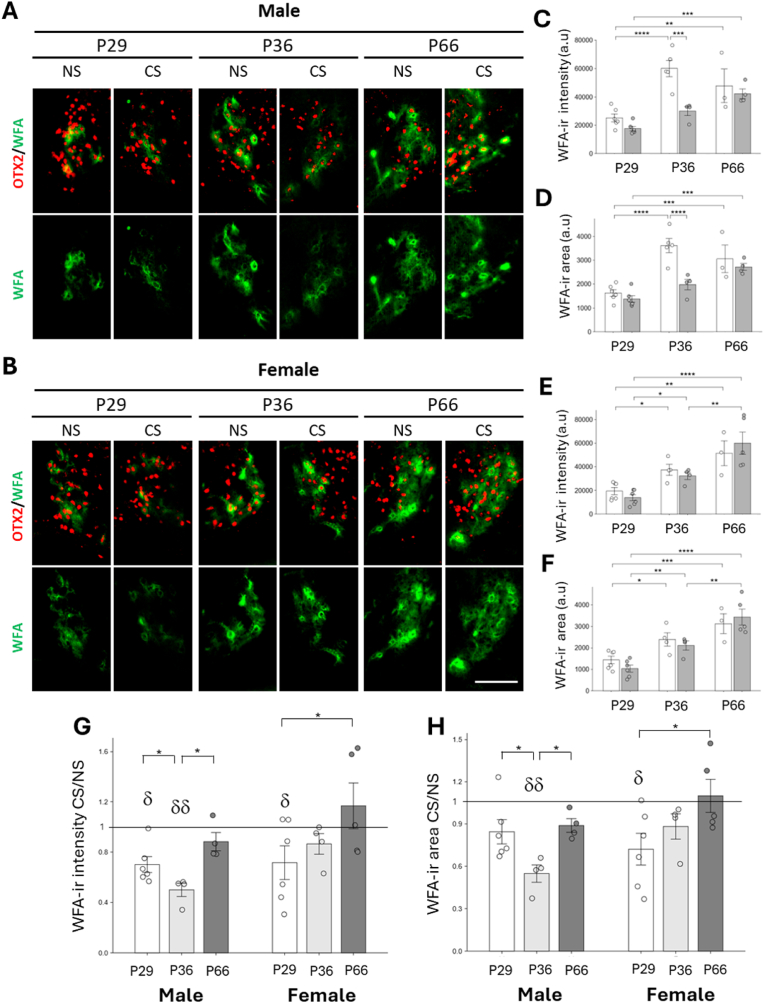
**Impact of chronic stress (CS) on PNNs in the IPN of male and female mice at different postnatal stages.** (A–B). Immunostaining of coronal sections in the lateralmost part of the lateral IPN (lat-cIPL) with anti-Otx2 (red) and WFA (green) in female (A) and male (B) mice subjected or not to chronic stress during P23–29, P30–36 and P60–66. Scale bar: 100 μm.(C–D) Quantification of WFA + immunoreactivity intensity (C,E) and area (D,F) in male (C–D) and female (E–F) mice at P29, P36, and P66. (G–H) quantification in C-F expressed as the ratio of chronic stress (CS) to no stress (NS). Quantifications under the NS condition are the same as those previously presented in Fig. 3. Number of male mice: P29: n = 6; P36: n = 4; P66: n = 5. Number of female mice: P29: n = 6; P36: n = 3; P66: n = 5. Error bars represent SEM. ∗P < 0.05; ∗∗P < 0.01; ∗∗∗P < 0.001; ∗∗∗∗P < 0.0001. Two-way ANOVA (D–F) and One-way ANOVA (G–H) and LSD (Least Significant Difference) test by Fisher as a post hoc test (D–H). ∗P < 0.05; Kruskal–Wallis and Mann-Whitney U as a post hoc test (H, female). ^δ^P< 0.05; ^δδ^P< 0.01. One sample *t*-test mean value = 1 (G–H).

Early life stress consistently leads to reductions in PNN density and integrity, particularly around PV + interneurons in many regions of the brain, including infralimbic cortex, prefrontal cortex, and amygdala (Santiago et al., 2018; Jakovljevic et al., 2022; Gildawie et al., 2021). We asked whether, in a similar way, chronic peripubertal stress could have an impact on the maturation of PNNs in the HIPOPS, and whether this impact affected males and females differently. To answer this question, we assessed the effects of chronic stress on PNN maturation by measuring the ratios of WFA intensity and area under chronic stress (CS) versus no stress (NS) conditions at postnatal stages P29, P36, and P66 (Fig. 4). The CS/NS ratio indicates the degree of influence of chronic stress on PNN development. The greater the impact of stress, the lower the ratio.

Analysis of WFA labeling revealed that stress impacted PNN development at distinct periods between male and female mice. In males, both the intensity and area of WFA labeling, expressed in absolute values, were significantly reduced in CS compared to NS controls specifically at P36, but not at P29 or P66 (Fig. 4A–C, D). When data were expressed as the CS/NS ratio, a significant decrease was again observed at P36 compared to P29 and P66 (Fig. 4G and H), indicating that chronic stress exerts its strongest effect on PNN density at this developmental stage. No significant differences were observed between P29 and P66.

Furthermore, the CS/NS ratios for both intensity and area of WFA labeling were significantly different from 1 at P36, confirming that CS has a robust effect at this time point. At P29, only the intensity ratio was significantly different from 1, suggesting that although the most sensitive window to stress-induced alterations in PNN density occurs at P36, a subtle effect may already be emerging as early as P29. These results suggest that, in males, P36 marks a critical period during which PNN development is particularly susceptible to modulation by chronic stress. In contrast, before (P29) and after (P66) this window, PNNs appear either less sensitive or more resistant to external stressors.

Females showed a different response profile. The intensity or area WFA labeling expressed as absolute numbers showed no significant decrease between CS and NS conditions at P36, or at other time points (Fig. 4B–E, F). Still, the CS/NS ratios for both intensity and area of WFA labeling at P29 were significantly lower than at P66 (Fig. 4D, ∗p < 0.05), indicating a stronger effect of chronic stress at P29. The difference between P36 and P66 was not significant, suggesting that the impact of chronic stress stabilizes after P29. Furthermore, the CS/NS ratios for both intensity and area of WFA labeling were significantly different from 1 only at P29. This pattern indicates that the period when PNN density is sensitive to the effects of chronic stress occurs earlier in females, i.e. at or before P29, and from P36 onwards, the impact of stress is very low, as observed in males at P66.

These results suggest that the periods during which chronic stress significantly influences PNN maturation differ between males and females. In males, the sensitive period appears to be at P36, a stage when PNN density is most affected by chronic stress before stress resistance develops at P66. In females, on the other hand, the sensitive period to the effects of chronic stress is much earlier, around P29. By P36, the effect of stress on PNN density has already diminished, indicating that the window of sensitivity closes earlier in females than in males.

5. PNN degradation renders the HIPOPS sensitive to stress in “closed” periods

**Fig. 5:**
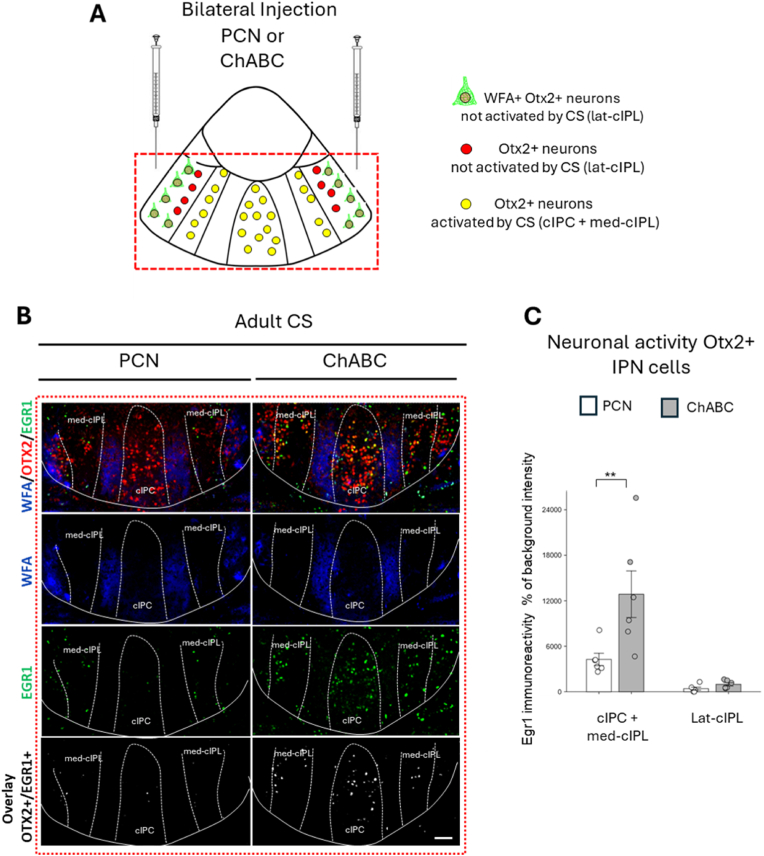
**Impact of PNN degradation on the stress response of Otx2^+^ neurons in the IPN of adult male mice.** (A) Schematic representation of a coronal section of the IPN, highlighting the cIPC + med-cIPL subregions containing CS-sensitive Otx2+ neurons (yellow) and the lat-cIPL subregions containing CS-insensitive Otx2+ neurons (red) and CS-insensitive Otx2+ neurons surrounded by PNNs (PNNs in green). The dashed red rectangle indicates the region shown in subfigures in B. (B) Immunostaining of coronal sections of the cIPN with anti-Otx2 (red), anti-Egr1 (green), WFA (blue), two weeks after bilateral injections of either penicillinase (PCN) or chondroitinase ABC (ChABC) into the cIPN, followed by exposure to chronic stress starting one week after surgery. The overlay between Otx2 and Egr1-immunoreactivities is shown (Bottom panels). Scale bar: 100 μm. (C) Quantification of Egr1 immunoreactivity in Otx2^+^ cells within the cIPC + medial cIPL and lateral cIPL regions in adult male mice that received bilateral stereotaxic injections of PCN (n = 7) or ChABC (n = 6) into the cIPN, followed by exposure to chronic stress starting one week after surgery. ∗∗P < 0.01; Mann-Whitney *U* test for comparison in cIPC + med-cIPL and two-tailed *t*-test for comparison in lat-cIPL.

Reduction of PNNs in response to stress has been shown to alter the electrical properties of parvalbumin(PV)-expressing neurons in the short and long term (Catale et al., 2022). We therefore asked whether PNN degradation might have an impact on how IPN neurons respond to chronic stress at a stage when IPN neurons typically do not respond to chronic stress. To directly test PNNs role in the stress response of Otx2+ neurons in the IPN, bilateral injections of chondroitinase-ABC (ChABC) or penicillinase (PCN) as a control solution were administered into the IPN of P53 male mice to achieve enzymatic digestion of PNNs (Fig. 5A). Adult chronic stress (P60-66) was then applied, and mice were sacrificed immediately after the last stress at P66. WFA analysis confirmed the efficiency of PNN degradation by ChABC (Fig. 5B).

We then assessed the impact of PNN degradation on the activity of Otx2+ IPN neurons in response to the adult chronic stress. Surprisingly, Egr1 labeling in the cIPC + med-cIPL regions (see Fig. 2A) increased significantly in ChABC-injected mice compared to PCN-injected mice (Fig. 5B–C). Egr1 immunoreactivity in the lat-cIPL (Fig. 5B and C) regions containing neurons surrounded by PNNs was low and did not differ significantly between PCN- and ChABC-treated mice.

These results demonstrate that PNN degradation enhances the stress response in Otx2+ neurons that are specifically responsive to stress during the early postnatal period. In adulthood, this neuronal population is typically not activated by stress, in the absence of prior peripubertal stress (Fig. 2B); however, when PNNs are degraded in the lat-cIPL region, neuronal activation is markedly enhanced by the adult stress, resembling the response observed following peripubertal stress exposure. This demonstrates that PNNs play a restrictive role in the stress response of Otx2+ neurons in the cIPC + med-cIPL regions, once the circuits have reached maturity. As the stress-responsive population is not the one surrounded by PNNs, this suggests that the lat-cIPL region exerts an inhibitory influence on mature cIPC and med-cIPL neurons.

6. PNN degradation reopens a critical period

The stress response critical period of HIPOPS is characterized by maximal induction of Otx2+ IPN neurons activity in male and female mice (see Fig. 2). Maturation of PNNs in the IPN occurs during this CP (see Fig. 3) and is only affected by stress when precisely applied during the CP, resulting in a decrease in PNN density (see Fig. 4). PNNs also appear to directly regulate the activity of Otx2+ IPN neurons. A reduction in PNN density induced by ChABC treatment in adulthood, a stage when stress does not activate the IPN, makes the IPN sensitive to stress (see Fig. 5). It is therefore possible that the state of PNN maturation is a key factor in stress inducing IPN hyperactivity and increasing susceptibility to anxiety (Fig. 1, Fig. 2).

**Fig. 6:**
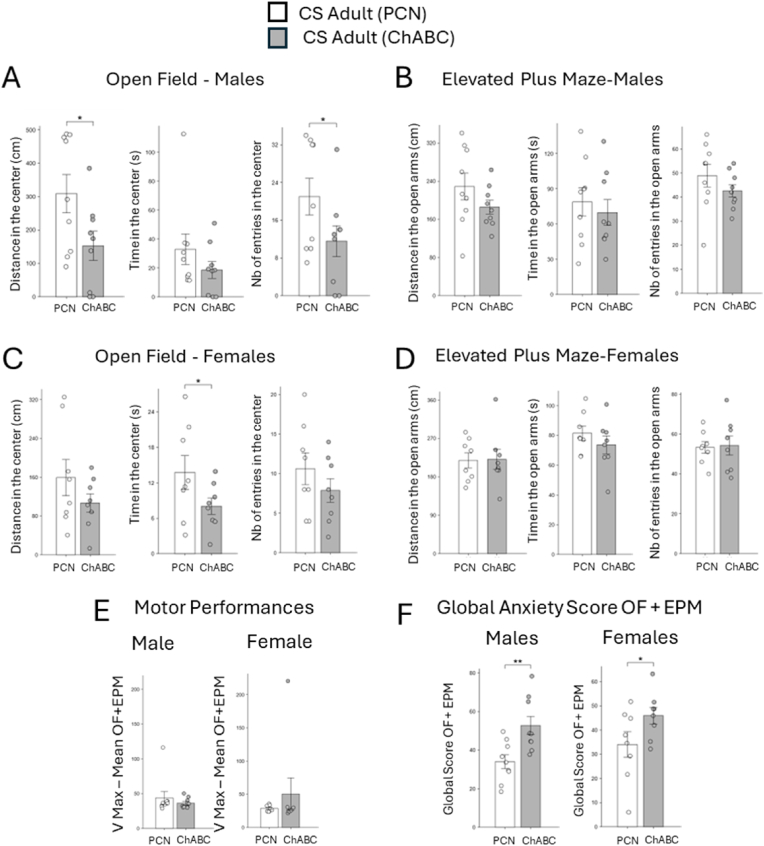
**Anxiety levels of male and female adult mice in OF and EPM behavioral tests 2 weeks after a chronic stress between P60 and P66**. (A–D) Comparison of anxiety levels in male and female mice injected with penicillinase or chondroitinase ABC (Male PCN, n = 9; Male ChABC, n = 9; Female PCN, n = 8; Female ChABC, n = 8) in the Open Field (A,C) and Elevated Plus Maze (EPM) tests (B,D). The following parameters were measured: Distance traveled, time spent and number of entries in the center (A,C) or in the open arms (B,D). Motor performance was assessed by averaging the maximum velocity measured in the OF and EPM tests (E). An anxiety score was calculated based on the different parameters (F). Data are presented as mean ± SEM. ∗P < 0.05; Mann-Whitney *U* test for comparison of distance in the center in (A) and ∗P < 0.05, ∗∗P < 0.01; two-tailed *t*-test for all other parameters. See Table 1 for statistical details.

We therefore tested whether enzymatic reduction of PNN density in adults could increase not only IPN sensitivity to stress (Fig. 5) but also reopen a state of susceptibility to stress-induced anxiety. To this end, bilateral injections of chondroitinase-ABC (ChABC) in the IPN of P53 mice were performed, with penicillinase (PCN) serving as a control. Mice were then exposed to chronic stress (P60-66). Four weeks after the stress period, allowing sufficient time for long-term plasticity mechanisms to take effect, behavioral analyses were performed using the Open Field (OF) and Elevated Plus Maze (EPM) tests (Fig. 6A–D). A global score combining the parameters from both tests was also calculated (Fig. 6F). A unilateral *t*-test was applied, justified by the prior hypothesis that PNN degradation would enhance anxiety, as previously associated with increased neuronal activation.

ChABC-injected male mice showed significantly higher anxiety-like behavior than PCN-injected controls, reflected by a decrease in the distance traveled and number of entries in the anxiogenic zone of the OF (Fig. 6A). All other parameters of anxiety-like behavior followed the same trend although not reaching statistical significance (Fig. 6A–B).

A similar trend was found in females, with ChABC-injected mice showing increased anxiety-like behavior compared to PCN-injected controls, revealed by significantly decreased time spent in the anxiogenic zone of the OF (Fig. 6C). Other parameters of the OF test followed the same trend although not reaching statistical significance. Results from the EPM showed no significant differences with only a modest reduced time spent in the anxiogenic zone following ChABC treatment (Fig. 6D).

A comprehensive analysis combining all parameters from both behavioral tests confirmed a significant increase in anxiety-like behavior following ChABC treatment in both male and female mice (Fig. 6F). Motor performance, evaluated by recording maximal speed of each animal in the 5-min tests, showed no difference between the two treatments, ruling out motor dysfunctions that could explain the variations identified in the tests (Fig. 6E).

Together, these results show that ChABC-mediated PNN degradation in the IPN of adult male and female mice reopens a critical period of sensitivity to chronic stress, potentiating anxiety-like behavior at a stage when animals are no longer durably affected by such a stress. Although the effects were less robust in female than in male mice, and not always significant at the level of individual parameters, the global analysis confirms the same overall trend in both sexes. This further supports the role of PNN surrounding 10.13039/501100003069IPN Otx2+ neurons in controlling the opening and closure of the HIPOPS critical period of sensitivity to chronic stress.

## 4 Discussion

In this study, we found that chronic peripubertal stress applied between P30 and P36, which strongly induces a high susceptibility to anxiety in male mice through hyperactivation of Otx2+ IPN neurons, has a much weaker effect in females. This correlates with a different reactivity profile of Otx2+ IPN neurons between males and females, with peak reactivity in females occurring a week earlier. Although the period of IPN plasticity, as evidenced by PNN maturation, appears similar in males and females, this PNN maturation process was differentially affected by stress, with females affected at an earlier period than males. Such a strong correlation between the effects of chronic stress on the activation of Otx2+ neurons of the IPN and on PNN maturation in both sexes suggests that the critical period of IPN sensitivity to stress occurs a week earlier in females than in males, and that in both sexes it is controlled by a sex-specific window of PNN maturation. In support of this hypothesis, we have shown that PNN degradation in the 10.13039/501100003069IPN of adult mice of both sexes reopens a period of high reactivity to chronic stress, leading to increased susceptibility to anxiety, even in the absence of peripubertal stress. This heightened anxiety susceptibility is associated with increased activity of IPN neurons, typically activated by peripubertal stress, suggesting that PNN degradation reopens a window of sensitivity to chronic stress in adulthood. However, it is important to note that the evidence of increased neuronal activity comes only from males, even though both sexes exhibit heightened anxiety following PNN degradation at the behavioral level.

Although Otx2+ neurons and the PNNs surrounding them are implicated in anxiety regulation, the precise mechanisms remain unclear. Notably, the population of neurons surrounded by PNNs marked by WFA shows only limited overlap with the population activated by chronic stress (Fig. 5A). This suggests that the effect of PNN degradation on neuronal activity in WFA-negative populations is likely indirect.

A plausible hypothesis is that Otx2+ neurons in the lat-cIPL normally limit stress-induced activation of Otx2+ neurons in the med-cIPL and cIPC regions via the habenula. This inhibitory function is fully effective when PNNs around lat-cIPL Otx2+ neurons are mature. However, stress disrupts this maturation, and consequently—or in parallel—induces activity in Otx2+ neurons of the med-cIPL and cIPC regions, leading to persistent circuit dysregulation, increased stress sensitivity, and greater susceptibility to anxiety. Supporting this hypothesis, a recent study has shown that neurons expressing DDR3 and showed to largely correspond to Otx2+ lat-cIPL are activated under anxiogenic environment to counteract anxiety (Handa et al., 2025). In this same study no projection from Drd3+ neurons to med-cIPL or cIPC neurons is described which would suggest that this inhibitory mechanism, if it exists, is likely indirect.”

In this scenario, fully mature PNNs serve as protective barriers, shielding circuits from stress-induced dysregulation. As a result, stress occurring after PNN maturation has less impact on neuronal circuits and anxiety. This could explain why stress applied at P30-36 in females has a reduced effect.

Additionally, we can predict that earlier stress at P23-29, which we showed affects PNN integrity and IPN Otx2+ neuronal activity in both males and females, would have a stronger and more prolonged impact in females than a stress applied at P30-36. The earlier closure of critical periods in females may be influenced by hormonal factors such as estrogen or progesterone. Such mechanisms could have evolutionary or adaptive significance in sex-specific stress responses. Future studies could explore how sex hormones modulate PNN maturation and whether manipulating hormone levels during puberty alters IPN response to stress and stress effect on long term anxiety. The observed variability in female anxiety responses highlights the need to investigate individual resilience factors. Examining genetic, epigenetic, and behavioral correlations of resilience could identify protective mechanisms that buffer against stress during critical periods.

Regarding the way PNN degradation impacts Otx2+ neuronal activity, it is important to note that not all PNNs are marked by WFA. For example, Aggrecan, another PNN marker, identifies Otx2+ neurons that are not labeled by WFA (Supplemental Fig. 2). This Aggrecan-marked population overlaps more closely with neurons activated by chronic stress and Aggrecan is also degraded with ChABC treatment (Supplemental Fig. 2), raising the possibility of a cell-autonomous effect of PNN degradation. Nonetheless, further studies are required to confirm this hypothesis.

Our findings suggest that PNNs are not merely passive stabilizers but play an active role in stress responses. Exploring the mechanisms by which PNNs are remodeled in response to stress or pharmacological interventions (e.g., ChABC treatment) could reveal novel therapeutic strategies for anxiety disorders. Identifying molecular players like Otx2 and Aggrecan in PNN dynamics will be critical. Indeed, Otx2 is a well-established regulator of PNN maturation, playing a crucial role in their development and maintenance. In other systems, this effect has been primarily attributed to exogenous Otx2. However, in the HIPOPS, Otx2 may originate from multiple sources, both exogenous and endogenous, adding complexity to its function. Notably, IPN neurons themselves express Otx2, further complicating its roles within these circuits and suggesting a more intricate interplay between intrinsic and extrinsic Otx2-driven mechanisms.

In our previous work (Rakotobe et al., 2024), we hypothesized that the deletion of Otx2 could influence the intrinsic activity of MHb neurons. However, we also proposed that a non–cell-autonomous mechanism might contribute to the observed phenotype. Specifically, Otx2 may normally be transferred from MHb terminals to the IPN, where it could facilitate the maturation of perineuronal nets (PNNs). Its absence may delay PNN development, thereby mitigating the long-term effects of chronic stress on IPN neurons and rendering them less responsive to future stressors. Our current findings lend support to this hypothesis, showing that PNNs regulate the chronic stress-induced activity of IPN neurons, an activity that, in turn, modulates anxiety levels. Nevertheless, this proposed mechanism requires further validation, including direct evidence that Otx2 is transferred to the IPN, that it contributes to PNN maturation, and electrophysiological studies assessing how neuronal excitability and synaptic function are affected when PNN integrity and Otx2 transfer are disrupted.

While the IPN is a key focus, PNNs are known to regulate plasticity in other regions implicated in stress and anxiety, such as the prefrontal cortex, amygdala, and hippocampus. Future studies could determine whether similar sex-specific patterns of stress susceptibility and PNN maturation exist in these regions and how they integrate with the HIPOPS.

In conclusion, this study reveals significant sex-specific differences in the sensitivity of PNNs and the HIPOPS system to peripubertal stress. The earlier sensitivity of PNNs to stress in females likely renders earlier stress more impactful in females than in males, whereas males exhibit a delayed onset of the stress-sensitive period relative to females. PNNs emerge as dynamic regulators of stress-induced neuronal activation and anxiety behaviors, providing a promising avenue for understanding and treating stress-related disorders. Future work should expand on the interplay between hormones, genetic factors, and PNN plasticity across brain regions and developmental stages to fully elucidate the mechanisms underlying stress resilience and vulnerability.

## CRediT authorship contribution statement

**Niels Fjerdingstad:** Writing – review & editing, Visualization, Methodology, Investigation, Formal analysis, Data curation. **Malalaniaina Rakotobe:** Writing – review & editing, Visualization, Methodology, Investigation, Formal analysis, Data curation. **Célia Leboulenger:** Methodology, Investigation. **Adrien Chopin:** Methodology, Investigation. **Thomas Lamonerie:** Writing – review & editing, Visualization, Validation, Supervision, Resources, Project administration, Funding acquisition, Data curation, Conceptualization. **Fabien D'Autréaux:** Writing – original draft, Visualization, Validation, Supervision, Project administration, Methodology, Investigation, Funding acquisition, Formal analysis, Data curation, Conceptualization.

## Declaration of competing interest

The authors have no conflicts of interest to declare.

## Data Availability

No data was used for the research described in the article.
