# Supplementary material for: Sexual dimorphism in anxiety susceptibility: role of PNN maturation timing in the habenulo-interpeduncular reward circuits
Source: Neurobiol Stress. 2025 Aug 17;38:100750. doi: 10.1016/j.ynstr.2025.100750 (PMC12395977; doi:10.1016/j.ynstr.2025.100750)
Supplement: Multimedia component 1 [file mmc1.docx]

Supplemental Figure 1


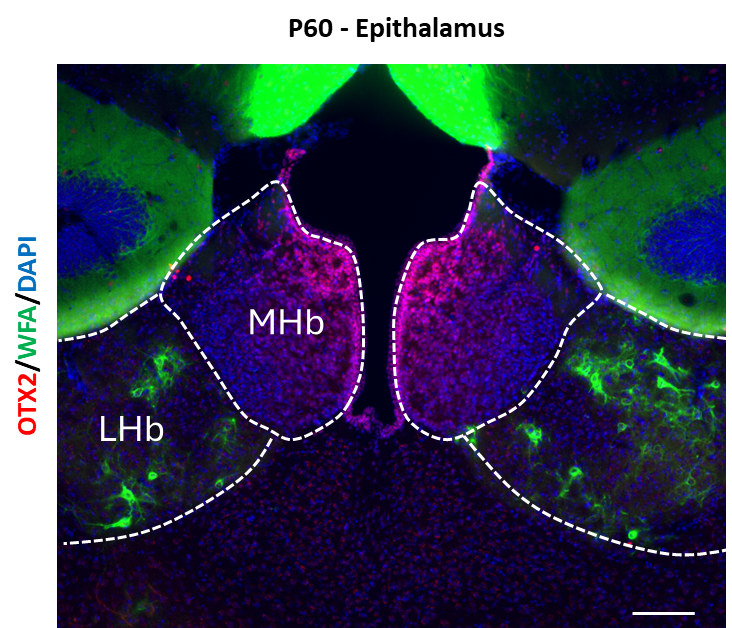


**Figure S1. Distribution of neurons surrounded by PNNs in the epithalamic region of adult mice.** Coronal section at the level of the habenula of young adult mice (P60; n=5). The Otx2^High^ region of the MHb, as described in ^12,13^, is Otx2-immunoreactive (red). WFA staining (green) is only detected in the lateral habenula (LHb), but not in the Medial habenula (MHb).

Supplemental Figure 2


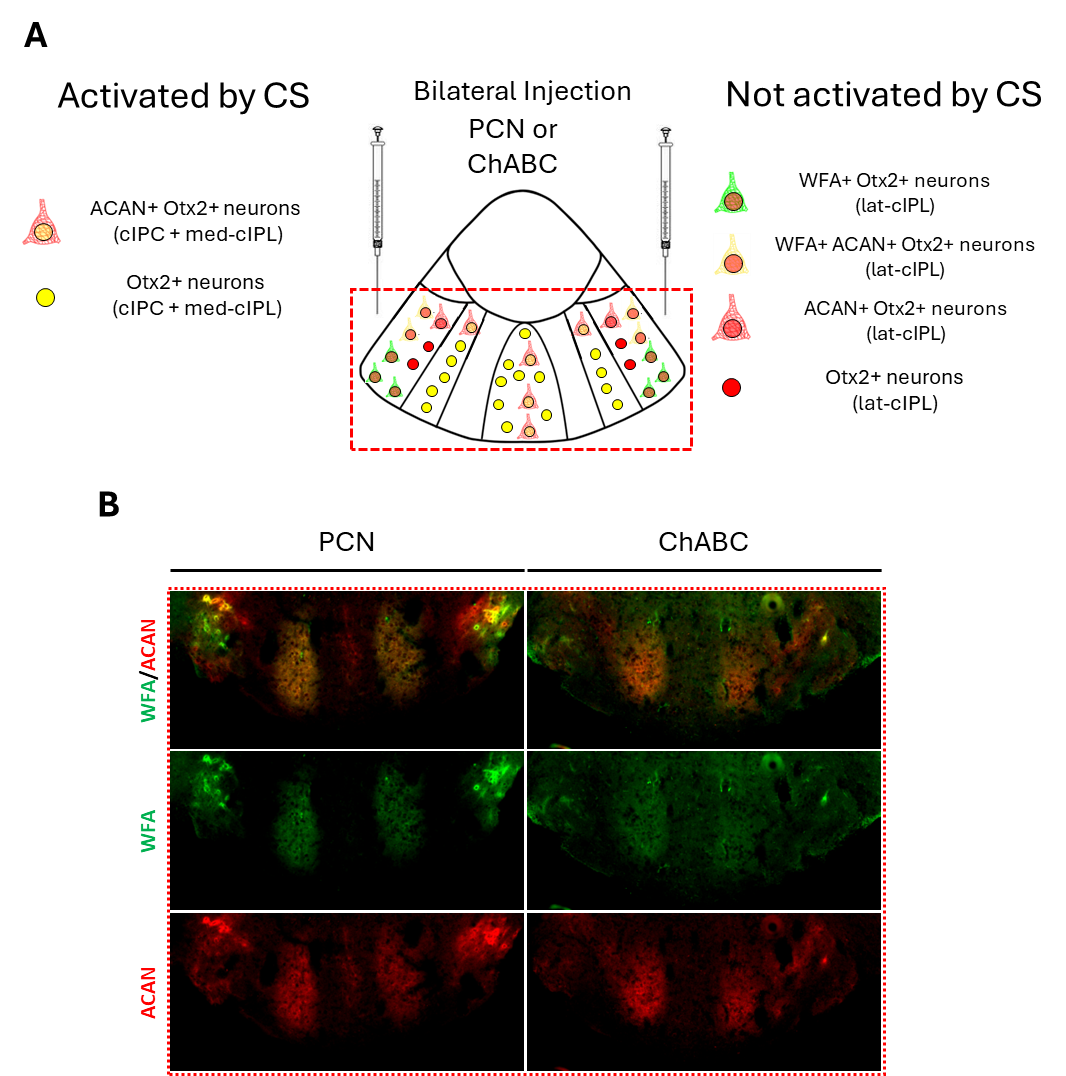


**Figure S2. Effect of chondroitinase ABC on PNNs labeled by WFA and/or containing Aggrecan in the IPN of adult male mice.** (A) Schematic representation of a coronal section of the IPN, highlighting the cIPC + med-cIPL subregions containing CS-sensitive Otx2+ neurons (yellow circle), CS-sensitive Otx2+ neurons surrounded by PNNs (yellow circle + red PNNs) and the lat-cIPL subregions containing CS-insensitive Otx2+ neurons (red), CS-insensitive Otx2+ neurons surrounded by PNNs labeled with WFA and Aggrecan (red circle + PNNs in yellow), CS-insensitive Otx2+ neurons surrounded by PNNs labeled with WFA (red circle + PNNs in green), CS-insensitive Otx2+ neurons surrounded by PNNs labeled with Aggrecan (red circle + PNNs in red). The dashed red rectangle indicates the region shown in subfigures in B. (B) Immunostaining of coronal sections of the cIPN with anti-Aggrecan (red), and WFA (green), two weeks after bilateral injections of either penicillinase (PCN) or chondroitinase ABC (ChABC) into the cIPN. Scale bar: 100 µm.
